# Laparoscopy-assisted Distal Gastrectomy should become a Standard Surgical Treatment Option for Stage IA/IB Gastric Cancer

**DOI:** 10.1055/s-0041-1725071

**Published:** 2021-03-16

**Authors:** Qian Liu

**Affiliations:** 1 Tianjin Lung Cancer Institute, Tianjin Medical University General Hospital, Tianjin, People's Republic of China

Gastric cancer (GC) is one of the most commonly occurring and deadly cancers in the world, 1 especially among Chinese. 2 3 In the early stages of GC, surgery with either subtotal or total gastrectomy, is the first treatment option. Current guidelines recommend open distal gastrectomy (ODG) with D2 lymph node dissection as the standard treatment for stage I GC. 4 5 However, laparoscopy-assisted distal gastrectomy (LADG), a minimally invasive technique, has become more widely adopted as it results in fewer blood loss, lower pain, and faster recovery than ODG. 6 7 Previous retrospective studies have reported there were no statistically significant differences in the overall survival of GC patients receiving LADG and those receiving ODG. 8 9 But selection bias might arise in these studies, because LADG is much more difficult than ODG. Therefore, it is necessary to determine whether LADG is good enough to replace ODG in the treatment of early-stage GC through randomized controlled, noninferiority clinical trials.

LADG has become one of the treatment options for stage I GC in the 2017 version of the Gastric Cancer Treatment Guidelines in Japan, 4 as the JCOG0703 10 and JCOG0912 11 clinical trials have confirmed its safety. However, several studies have reported that cancer cells can be recovered in the exhaust gas and instruments used in laparoscopy. 12 13 These findings suggest that laparoscopy may increase the risk of cancer cells spreading. Thus, the long-term efficacy of LADG is still controversial. In a study recently published in *Lancet Gastroenterology & Hepatology* , titled “Survival outcomes after laparoscopy-assisted distal gastrectomy versus open distal gastrectomy with nodal dissection for clinical stage IA or IB gastric cancer (JCOG0912): a multicentre, non-inferiority, phase 3 randomised controlled trial,” 14 JCOG0912 team led by Prof. Hitoshi Katai from the National Cancer Center Hospital in Japan reported the final results of the long-term survival data in JCOG0912 trial to determine if LADG was noninferior to ODG.

A total of 921 patients with stage I GC in this study were enrolled between March, 2010 and November, 2013. They were randomly assigned in a 1:1 ratio to receive LADG ( *n* = 462) or ODG ( *n* = 459) at 33 institutions in Japan. Among them, 99% ( *n* = 912) patients successfully received the designated surgery. The 5-year relapse-free survival (RFS) was 95.1% (95% confidence Interval [CI] = 92.7–96.8%) in the LADG group, while that was 94.0% (95% CI = 91.4–95.9%) in the ODG group. LADG was noninferior to ODG for RFS (hazard ratio [HR] = 0.84; 90% CI = 0.56–1.27; *p* = 0.0075). No patient died because of the surgical treatment. The most commonly occurring postoperative adverse event (AE) was bowel obstruction. There were 5 (1.09%) patients in the LADG group and 11 (2.42%) in the ODG group who developed grade 3 or 4 bowel obstruction.

In the previous report of JCOG0912 trial, 11 Prof. Katai and colleagues demonstrated that the frequency of AEs and short-term clinical outcomes of the early-stage GC patients in LADG group were comparable to those in ODG group, but patients receiving LADG lost less blood, recovered faster, and used fewer analgesic doses after surgery than those receiving ODG. Their findings are consistent with the KLASS-01 trial conducted in Korea. 15 Moreover, the long-term data in this study demonstrated that the RFS of the patients treated with LADG is noninferior to those with ODG. The KLASS-01 trial also supports this conclusion. 15 The advantages of this study are as follows: (1) compared with the KLASS-01 trial, this study has a lower proportion of ineligible patients (4% in the KLASS-01 trial, while < 1% in this study) and fewer patients who have not undergone designated surgery (6% in the KLASS-01 trial, while 1% in this study); (2) participants in this study have higher adherence to surgical assignment; (3) under the guidance of the Japan Clinical Oncology Group (JCOG), which is proficient in conducting clinical trials, all details of the JCOG0912 protocol were strictly followed during the study period; (4) due to the high incidence of GC in Japan, Japanese doctors have rich clinical experience in diagnosing and treating GC, so the diagnostic accuracy (4.4% of the study population was found to have higher disease stages than stage I) and the levels of surgical technology in this study are currently the highest in the world. However, this clinical trial has several limitations: (1) the noninferiority HR is 1.54, which is slightly higher than usual in clinical studies; (2) only enrolled stage I GC patients treated by distal gastrectomy; (3) there were approximately 25% patients enrolled in this study received pylorus-preserving gastrectomy (a medical operation that modifies the infrapyloric node dissection and omits the suprapyloric lymph node dissection); (4) the upper limit of CI did not exceed the noninferiority margin, but the CI of HR was wide; (5) it is unclear whether the findings can be applied to non-Japanese patients and used to predict the outcomes of LADG and ODG performed by non-Japanese surgeons.

Despite these limitations, this open-label, multicentre, phase III randomized controlled trial has more accurately evaluated if LADG was noninferior to ODG in the treatment of early-stage GC. The findings suggest that LADG should become a standard surgical treatment option for stage I GC. In future clinical trials, the efficacy and safety of LADG for total gastrectomy and for more advanced GC must be accessed.
